# Cardiovascular Complications of Sleep Apnea: Role of Oxidative Stress

**DOI:** 10.1155/2014/985258

**Published:** 2014-03-06

**Authors:** Mohammad Badran, Najib Ayas, Ismail Laher

**Affiliations:** ^1^Department of Pharmacology and Therapeutics, Faculty of Medicine, University of British Columbia, Vancouver, BC, Canada V6T 1Z3; ^2^Divisions of Critical Care and Respiratory Medicine, Department of Medicine, Sleep Disorders Program, UBC Hospital, Division of Critical Care Medicine, Providence Health Care, University of British Columbia, Canada V6Z 1Y6

## Abstract

Obstructive sleep apnea (OSA) occurs in 2% of middle-aged women and 4% of middle-aged men with a higher prevalence among obese subjects. This condition is considered as an independent risk factor for cerebrovascular and cardiovascular diseases. One of the major pathophysiological characteristics of OSA is intermittent hypoxia. Hypoxia can lead to oxidative stress and overproduction of reactive oxygen species, which can lead to endothelial dysfunction, a hallmark of atherosclerosis. Many animal models, such as the rodent model of intermittent hypoxia, mimic obstructive sleep apnea in human patients and allow more in-depth investigation of biological and cellular mechanisms of this condition. This review discusses the role of oxidative stress in cardiovascular disease resulting from OSA in humans and animal models.

## 1. Introduction

Obstructive sleep apnea (OSA) is a sleep-breathing disorder characterized by intermittent episodes of either complete breathing cessation for periods of ten seconds or more (apnea) or significant reductions in breathing amplitude (hypopnea). Patients are categorized as having mild, moderate, or severe OSA depending on the apnea/hypopnea index (AHI), which is the total number of apnea/hypopnea episodes per hour of sleep. In normal individuals the index is usually 5 or lower, 5–15 in mild, 15–30 in moderate, and 30 or more in severe OSA patients [1]. The severity of OSA is accompanied by significant episodes of hypoxemia and hypercapnia, where in mild patients the oxyhemoglobin saturation drops to 95% and can drop below 80% in severe cases. Risk factors for sleep apnea include obesity, craniofacial abnormalities, smoking, male gender, short neck, and menopause in women. Obesity is one of the main risk factors of sleep apnea since 60% to 90% of OSA patients are obese and there is a strong positive correlation between BMI and OSA [2]. The overlap of obesity and OSA makes the identification of OSA versus obesity contributions to cardiovascular risk more challenging. Increased adiposity and short neck add weight to the soft tissue volume within the upper airway and the neck, further increasing airway collapsibility [3].

At a population level, at least 4% males and 2% females are diagnosed with sleep apnea and its symptoms, and it is estimated that 1 of every 5 adults have OSA and that 1 of every 15 adults have moderate OSA. In females, the prevalence increases from 3% for the third decade of life to 36% in the seventh decade. In men the prevalence for the third decade is 4% and increases to 50% during the seventh decade [15]. Unfortunately, most of those who are affected by OSA remain undiagnosed despite medical advances. Polysomnography is the main tool for diagnosing patients with OSA where sleep stages are monitored along with arterial blood gases, breathing, and electrocardiogram.

The results of many clinical studies strongly suggest that OSA is an independent risk factor for cardiovascular diseases such as hypertension, coronary artery disease, stroke, and heart failure. Several mechanisms have been suggested to link OSA and vascular diseases; evidence for this includes increases in sympathetic activation, oxidative stress, inflammation, endothelial dysfunction, coagulation, and metabolic dysregulation [16]. This review describes the role of free radicals and oxidative stress in cardiovascular disease in OSA patients and animal models of sleep apnea.

## 2. Cardiovascular Consequences of Sleep Apnea

Many of the reports correlating OSA to vascular disease come from small longitudinal studies of incidental cardiovascular disease and studies evaluating the effect of CPAP intervention. However, largely due to the cost of OSA diagnosis in large population samples, many studies can only indirectly implicate OSA in the etiology of cardiovascular disease. In addition, comorbidities such as obesity and hypertension that coexist with the majority of OSA patients make the independent risk of OSA on vascular disease more difficult to assess.  Table 1 summarizes some cohort studies relating OSA and incidence of cardiovascular disease.

### 2.1. Hypertension

Close to 35% of OSA patients have hypertension while 30% of hypertensive patients who have OSA are undiagnosed [17, 18]. In the Wisconsin Sleep Cohort, subjects with an AHI of 15 or higher had an almost 3-fold increased risk of developing hypertension when compared to control after 4 years [4]. On the other hand, the Sleep Heart Health Study (SHHS) reported that the unadjusted risk of hypertension increased with the severity of OSA after a 5-year followup, but this association was not significant after adjustment for body mass index [19]. This discrepancy in findings could be related to differences in the study sample characteristics and the techniques used to diagnose OSA. However, a recent study by Pedrosa et al. shows that OSA is the most common condition associated with resistant hypertension (64%), followed by primary aldosteronism (5.6%) and renal artery stenosis (2.4%) in 125 patients with resistant hypertension [20]. OSA is now included as one of the main causes of hypertension in the seventh report of the Joint National Committee on Prevention, Detection, Evaluation, and Treatment of High Blood Pressure [21]. Nasal continuous positive airway pressure (nCPAP) treatment reduces mean arterial blood pressure by 10 mm Hg in OSA patients both at night and day, a benefit that was lost when nCPAP treatment was subtherapeutic [22].

### 2.2. Coronary Artery Disease

The prevalence of OSA in patients with coronary artery disease is around 30% [23], while the prevalence among men hospitalized with acute myocardial infarction is nearly 70% [24]. Many characteristics of OSA can lead to cardiac ischemia such as intermittent hypoxia, sympathetic vasoconstriction and increased intrathoracic pressure. Sorajja et al. studied 200 subjects without a history of coronary artery disease and found the median coronary artery calcification score (Agatston units) was 9 in OSA patients compared to non-OSA patients. They also found that the median calcification score increased with the severity of OSA [25]. Another study shows that OSA patients are more likely to have a family history of premature death from coronary artery disease. Those results were independent of BMI, gender, and personal history of coronary artery disease [26]. However, a longitudinal analysis of data from the SHHS found that OSA was not a significant predictor of incidental CAD after adjustment for other risk factors [5].

### 2.3. Stroke

The prevalence of OSA in patients who have had a stroke is nearly 60% [27]. In a cross-sectional study of over 6,000 subjects from SHHS, the odds ratio of prevalent stroke was higher (1.58) in OSA patients with AHI ≥ 11 [28]. Several observational studies also show that OSA increases the chance of stroke incident [6, 29]. Redline et al. followed a total of 5,422 participants without a history of stroke at the baseline examination and untreated for OSA for a median of 8.7 years. A significant positive association between ischemic stroke and AHI was detected in men (*P* = 0.016). In OSA patients with AHI of 5–25, each unitary increase in AHI severity was associated with a 6% increase in stroke risk [30]. However, some factors rendered the relationship between OSA and stroke circumstantial. The population of patients who are at risk of stroke is demographically similar to the patients who are at risk of OSA. Also the fact that only survivors of stroke are tested complicates the causal association between stroke and OSA [31].

### 2.4. Heart Failure

The prevalence of OSA in HF ranges from 12% to 53% [32, 33]. Having OSA increases the mortality risk of patients with ischemic HF, mainly because of sudden death [33]. Wang et al. showed that untreated OSA (AHI ≥ 15) in HF patients was associated with increased mortality rates when compared to (AHI ≤ 15) [34]. Gottlieb et al. reported that OSA predicted incident heart failure in men but not in women (adjusted hazard ratio 1.13 per 10-unit increase in AHI). Men with AHI > or = 30 were 58% more likely to develop heart failure than those with AHI < 5 [5]. OSA can lead to heart failure through many mechanisms including increases in blood pressure, left ventricular afterload, and by greater risk of myocardial infarction [35]. It is clear that OSA is related to many cardiovascular diseases and its treatment is a necessity.

## 3. Oxidative Stress in OSA

Oxidative stress results from an imbalance between antioxidant defense mechanisms and the production of oxidants; meaning that either a decreased antioxidant capacity and/or overproduction of reactive oxygen and nitrogen species (ROS/RNS) leads to a state of oxidative stress (Table 2). Although free radicals have important roles in regulating signal transduction and cellular function, their overproduction can damage lipids, proteins, and DNA, thus affecting many cellular and physiological mechanisms. Recent studies show important links between the hypoxia-related free radicals related oxidative stress and cardiovascular disease in OSA patients.

### 3.1. Sources of Reactive Oxygen Species in OSA

Free radicals are atoms or molecules containing one or more unpaired electrons in their atomic or molecular orbitals and thus are chemically unstable and highly reactive. Usually when two radicals react, the product is a nonradical, but when radicals react with nonradicals the product is a new radical and, therefore, the radical chain reaction propagates [36]. Oxygen metabolism during normal cellular respiration generates ROS as by-products, and their elimination occurs through enzymatic and nonenzymatic antioxidant systems. When ROS generation exceeds the capacity of antioxidants, oxidative stress and damage to cells and tissues ensue. This can contribute to pathological conditions of cardiovascular disease.

Oxygen has a unique electronic configuration; the addition of one electron to molecular oxygen can result in the production of the superoxide anion. Superoxide is considered the primary ROS and can interact with other molecules to generate secondary ROS/RNS, either directly or through enzyme- or metal-catalyzed processes. Superoxide anions can give rise to the production of many toxic molecules such as hydrogen peroxide, hydroxyl radical and peroxynitrite [37]. The latter is a RNS and results from a reaction between superoxide anion and nitric oxide, an important endothelium-derived vasodilator. As a result, nitric oxide bioavailability decreases and the vasodilator ability of blood vessels is compromised [38].

**Figure 1:**
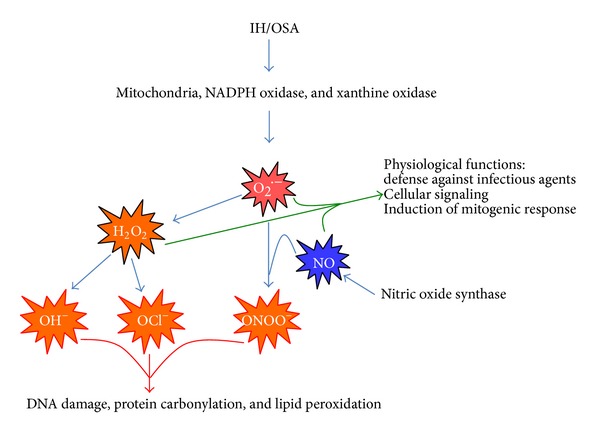
Reactive oxygen/nitrogen species produced during OSA/IH. H_2_O_2_: hydrogen peroxide, IH: intermittent hypoxia, NADPH oxidase: nicotinamide adenine dinucleotide phosphate oxidase, NO: nitric oxide, O_2_^−^: superoxide anion, OCl^−^: hypochlorite anion, OH^−^: hydroxyl anion, ONOO^−^: peroxynitrite, OSA: obstructive sleep apnea.

Mitochondria are major sources of superoxide anion due to reactions occurring during oxidative phosphorylation. It is estimated that 3–5% of the oxygen consumed by mitochondria is converted to superoxide anion during aerobic respiration. During hypoxia, ROS production is elevated due to excessive mitochondrial reduction [36]. NADPH oxidase is also a very important source of superoxide anion. Phagocytic cells contain this enzyme and other enzymes to produce ROS as a defense mechanism against pathogens. Although this mechanism can protect against invading microbes, it can also cause damage to surrounding tissue [39]. NADPH oxidase is also expressed in nonphagocytic cells where it usually generates lower amounts of superoxide anion for purposes such as signaling [40]. For example, NADPH oxidase is expressed in vascular cells where generation of superoxides plays an important role in vascular cell growth [41].  Figure 1 shows the production of different ROS, their physiologic function and role in disease state.

### 3.2. Evidence of Oxidative Stress in OSA

Many studies confirm the association of OSA with oxidative stress through measurements of oxidative stress markers. For example, Schulz et al. report increased production of superoxide anion in stimulated neutrophils and monocytes from OSA patients [42], while others report that superoxide anion production was significantly higher in nonstimulated monocytes of OSA patients [43, 44].

Oxidative stress markers of lipid peroxidation, protein carbonylation and DNA oxidation are increased in OSA patients. Lipid peroxidation is an important marker of oxidative stress since lipids are easily oxidized. Many studies show that lipid peroxidation increases in OSA patients. In an overnight study of OSA patients with and without cardiovascular disease, levels of thiobarbituric acid (TBARS), a marker of lipid peroxidation, were significantly increased [45]. In another study, fourteen males with severe OSA fasted all night and TBARS levels were measured in the next morning. TBARS levels in those patients were significantly higher (28.1 nmol MDA·mg^−1^ LDL protein) compared to thirteen healthy age matched controls (20.0 nmol MDA·mg^−1^ LDL protein) [46]. Oxidized LDL is also increased in OSA, where plasma levels of oxidized LDL were higher in OSA patients (43.6 U/L) compared to control (32.3 U/L) [47]. Protein carbonylation (oxidation of protein side chain) is increased as well in patients with moderate to severe OSA where protein carbonyl levels were significantly higher (1.11 *μ*mol/g protein) when compared to matched controls (0.99 *μ*mol/g protein). On the other hand, the increase was not significant in mild OSA patients (1.03 *μ*mol/g protein) [48]. 8-hydroxyl-2′deoxyguanosine (8-OHdG), a marker of DNA oxidation, is also elevated in OSA patients. Urinary excretion of 8-OHdG significantly correlates with the severity of OSA [49].

Evaluating oxidative damage in OSA patients is essential since oxidative stress is one of the main causes of endothelial dysfunction. Yamauchi et al. studied 32 OSA and 15 control patients, in which they quantified endothelial nitric oxide synthase (eNOS), phosphorylated eNOS (the active form of the enzyme responsible for producing NO in the vasculature), inflammation (cyclooxygenase-2 and inducible NOS), and oxidative stress (nitrotyrosine). They also evaluated vascular reactivity in these patients by flow-mediated dilation [49]. Endothelial expression of eNOS and phosphorylated eNOS decreased by 59% and 94%, respectively, in untreated OSA patients. Nitrotyrosine and cyclooxygenase-2 expression was 5-fold greater in OSA patients. In patients who adhered to CPAP ≥ 4 hours a day, the expression of nitrotyrosine, cyclooxygenase-2, and inducible NOS was decreased significantly, while CPAP treatment restored eNOS and phosphorylated eNOS expression levels with concomitant reduction in oxidative stress. Of interest is that the effect of CPAP may be restricted to limiting free radical production, as antioxidant defense mechanisms were unaffected. Flow-mediated dilation in OSA patients was significantly decreased, but adhering to CPAP ≥ 4 hours a day significantly improved endothelial dependent vasodilation [50].

Antioxidant capacity is impaired in OSA patients. Although the antioxidant capacity in OSA subjects and controls did not differ in their study, Christou et al. showed a linear negative relationship between antioxidant capacity and apnea/hypopnea index (*R* = −0.551, *P* = 0.041) [51]. Total antioxidant status in OSA patients is significantly decreased (1.4 mmol/L) when compared to healthy subjects (1.5 mmol/L, *P* = 0.0001), with lower levels of vitamin A (64 *μ*g/dL) and vitamin E (1525 *μ*g/dL) when compared to control (74 and 1774 *μ*g/dL, resp.) [52]. On the other hand, Katsoulis et al. reported some unexpected results where they found that total antioxidant status before and after sleep was significantly lower in OSA patients with AHI < 30 (1.73 versus 1.65 mmol/L, *P* = 0.01) but not in severe OSA patients with AHI > 30 (1.64 versus 1.58 mmol/L, *P* = 0.07). A possible explanation could be due to differences between the acute effects of hypoxia resulting from apneic sleep and chronic oxidative stress that may be sustained in severe OSA patients even during the daytime [53].

### 3.3. Oxidative Stress in Animals Subjected to Intermittent Hypoxia

OSA patients usually have comorbidities such as obesity, diabetes, or hypertension that likely will affect cause-effect relationships. Creating animal models of OSA would minimize the influence of comorbidities and behavioral variables common in humans. Using animal models also permits the use of pharmacological agents to study the pathological mechanisms under a well-controlled environment. Ideally, animal models should mimic OSA in humans in at least three ways: (a) they share aspects of the underlying pathophysiology, (b) have similar symptoms and the spectrum of disease severity that occur in humans, and (c) respond to treatment modalities that are useful in humans. Furthermore, a short life span (to allow for the unveiling of a wide range of disease-related complications within a reasonable time period), routine availability, cost effectiveness, and availability of disease-free littermates add to the usefulness of animal models. There are additional considerations when using animals that need to be considered for sleep-related research. Animal models for studying sleep-disordered breathing should address at least one (or a combination) of the three main injurious consequences of sleep apnea: intermittent hypoxia/hypercapnia, strained breathing due to mechanical obstruction, and sleep fragmentation. In this regard, rodents are amenable to genetic manipulation suitable for the production of phenotypes that may characterize OSA in humans. One advantage of using rodent models to examine neurophysiological aspects of sleep apnea in humans is the high degree of similarity between the structures of the nervous systems of rodents, such as rats and mice and humans.

A useful animal model of OSA is the English bulldog, since no surgical interventions or genetic manipulations are required. There is a strong resemblance in sleep apnea between humans and English bulldogs, making this animal model a suitable candidate for experimental use. It was noticed that these dogs snore and have hypopneas and frequent arousals from sleep, mainly due to an abnormal upper airway anatomy characterized by an enlarged soft palate and a narrowing of the oropharynx. These animals have episodes of both central and obstructive apnea with hemoglobin desaturation (<90%) that worsens during rapid eye movement (REM) sleep and is accompanied by daytime hypersomnolence as evidenced by a shortened sleep latency [54].

Alterations in the contractility of respiratory muscles were first reported in genetically obese Zucker fat rats (ZFR) in 1996 [55]. These animals show many of the cardiopulmonary deficits described in obese humans, such as respiratory control dysfunction, chest wall limitation, upper airway narrowing, hypertension, myocardial hypertrophy, and poor exercise capacity [56–58]. Later studies suggested that these rats also exhibit signs of sleep apnea [59].

OSA can also be stimulated through surgical procedures that induce airway obstruction [60]. This procedure has mainly been used in larger animals such as dogs Katayama et al. and Kimoff et al. [60, 61], piglets [62], baboons [63], and small rodents [64, 65]. Studies have incorporated sophisticated apparatus to detect sleep-wake states so that initiation of airway obstruction could be coordinated with sleep onset [66].

The most commonly used animal model in the area of OSA is the intermittent hypoxia (IH) model. This murine model represents extreme physiological changes occurring during sleep-related IH and was first described in 2001 by Tagaito et al. [67]. Mice are housed in customized cages to deliver either an intermittent hypoxic stimulus or an intermittent room air control. Ports evenly spaced near the bottom of the cages allow gases to enter from four sides at the level of the bedding material. A gas control delivery system regulates the flow of room air, N_2_, and O_2_ into the customized cages housing the mice. Programmable solenoids and flow regulators control the manipulation of inspired O_2_ fraction (FI_O_2__) levels in each cage over a wide range of IH profiles. During the 12-h light cycle, FI_O_2__ is reduced from 20.9 to 5.0% over a 30-s period and rapidly reoxygenated to room air levels using a burst of 100% O_2_ during the following 30-s period. During the 12-h dark cycle, a constant flow of room air is delivered to the cages. The use of multiple inputs into the cage produces a uniform nadir FI_O_2__ level throughout the cage.

There is much support in the literature for the idea that oxidative stress is a consequence of intermittent hypoxia. Rats subjected to intermittent hypoxia for two weeks have increased vascular production of ROS [13]. IH-induced pulmonary hypertension in mice leads to increased lung levels of the NADPH oxidase subunits NOX4 and p22phox, indicating that NADPH oxidase-derived ROS contributes to the development of pulmonary hypertension caused by chronic intermittent hypoxia [68]. NADPH oxidase is activated in tissues such as the myocardium, brain, carotid body, and liver in various animal models of IH [69–71]. As for oxidative stress markers, one month of IH significantly increased MDA levels in mice [8]. This is in agreement with a study by Savransky et al. who reported that serum MDA levels increased 4-fold in mice subjected to chronic IH for 6 months when compared to control [72]. Oxidative stress markers are also elevated in tissues such as the liver and brain Rosa et al. and Xu et al. [11, 73].

### 3.4. Oxidative Stress Causes Endothelial Dysfunction in OSA

Diminished endothelial function is an important consequence of OSA and is frequently measured as impaired endothelium dependent vasodilatation [74]. Eventually endothelial dysfunction leads to atherosclerosis, a condition where artery walls become narrow due to the buildup of fatty materials, cholesterol, macrophages, cellular debris, and other substances. These changes create significant reductions in blood flow through the affected artery [75]. Although the etiology of atherosclerosis is unknown, several factors such as elevated levels of LDL, low levels of HDL, hypertension, diabetes mellitus, male gender, obesity, family history, infectious disease, and environmental factors are implicated. Many of these factors lead to endothelial dysfunction and atherosclerosis through a unifying mechanism of oxidative stress and inflammation [76]. Various studies show lower levels of circulating NO in OSA, for example, by the reduced levels of serum nitrite/nitrate (by-products of normal NO metabolism) in OSA subjects (38.9 *μ*M versus 63.1 *μ*M in controls) [77]. This was confirmed in other studies where nitrate/nitrite levels were significantly lower in OSA patients (35.6 *μ*M) when compared to control (72.6 *μ*M) [78]. Many mechanisms have been suggested for endothelial dysfunction due to OSA or IH including (1) interaction on NO and ROS forming peroxynitrite, (2) uncoupling of eNOS, and (3) decreased endothelial expression of eNOS and increased levels of endogenous eNOS inhibitors [42]. Due to its short half-life and large volume of distribution, peroxynitrite is hard to measure and these factors explain the lack of difference in nitrotyrosine levels between OSA and healthy subjects [79, 80]. However, Jelic and Le Jemtel found an increased expression of nitrotyrosine in endothelial cells derived from OSA patients [50].

**Figure 2:**
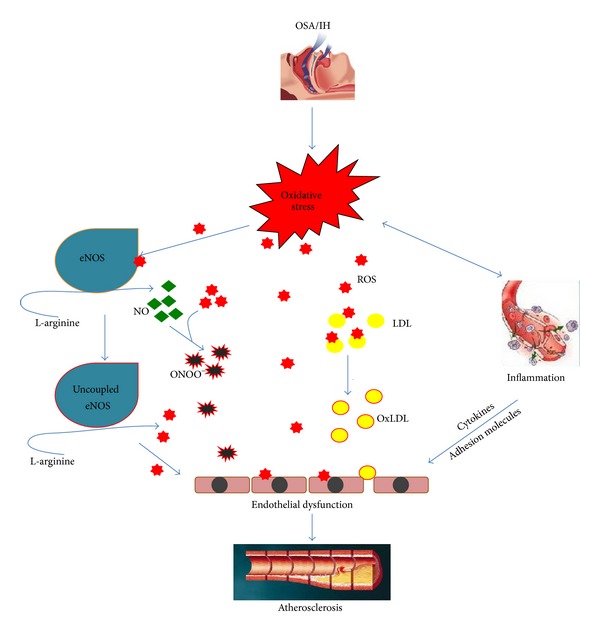
OSA/IH can lead to oxidative stress, which through many mechanisms can cause endothelial function, which eventually progresses to atherosclerosis. eNOS: endothelial nitric oxide synthase, IH: intermittent hypoxia, LDL: low density lipoprotein, NO: nitric oxide, ONOO^−^: peroxynitrite, OSA: obstructive sleep apnea, OxLDL: oxidized low density lipoprotein, ROS: reactive oxygen species,.

In all the forms of nitric oxide synthase, including the endothelial one, enzymatic activity requires five cofactor groups to incorporate oxygen into the amino acid L-arginine to produce NO. Those cofactors are flavin adenine dinucleotide (FAD), flavin mononucleotide (FMN), heme, tetrahydrobiopterin (BH_4_), and Ca^2+^-calmodulin. If nitric oxide synthase lacks L-arginine or another of the necessary cofactors, it will produce superoxide anion instead of NO through an uncoupled state of nitric oxide synthase [81]. Antoniades et al. reported that increased ROS production during hypoxia could lead to BH_4 _oxidation and increased levels of arginase II that degrades L-arginine, leading to further eNOS uncoupling [82]. Patients with OSA have increased levels of asymmetrical dimethylarginine (ADMA), a competitive inhibitor of NOS [83]. Studies by Tanaka et al. suggest that eNOS activation is sensitive to regulation by redox status and that oxidative stress leads to decreased eNOS phosphorylation, so reducing its activity [84], while Jelic and Le Jemtel supported the latter findings when they reported decreased ratios of total phosphorylated eNOS in endothelial cells from OSA [50].  Figure 2 explains how OSA can lead to atherosclerosis through oxidative stress mediated mechanisms.

### 3.5. Oxidative Stress and Heart Failure in OSA

Almost 20–40% of OSA patients have mild pulmonary hypertension even in the absence of lung disease or left-sided heart disease [85–87]. Severe OSA patients also seem to have abnormalities of right ventricular function [88–90]. The clinical significance of pulmonary pressure changes and right ventricular function is uncertain, and it is not known whether they are sufficient to progress to right ventricular failure in the absence of other cardiopulmonary diseases. It seems that OSA can lead also to left ventricular heart failure when there are comorbidities such as chronic lung disease, obesity, or left ventricular failure [91].

During hypoxic conditions, pulmonary artery constricts mainly due to the ability of their smooth muscles to sense changes in O_2_. An increase in pulmonary vascular resistance exerts a pressure overload to the right ventricle, resulting in hypertrophy followed by dilated cardiomyopathy [92]. In addition to the pulmonary artery, the carotid body can also sense changes in O_2_. It is well established that oxygen sensing by the carotid body plays an important role in the development of systemic hypertension associated with intermittent hypoxia and OSA [93]. The molecular mechanisms of oxygen sensing in these arteries involve ROS-induced closure of K^+^ channels which might be responsible for the acute changes in response to altered oxygen tension [94].

## 4. Summary

Obstructive sleep apnea is an independent risk factor for cardiovascular disease. It is well accepted that intermittent hypoxia in OSA resembles hypoxia/reperfusion injury mechanisms responsible for ROS overproduction. Intermittent hypoxia can promote endothelial dysfunction and heart disease through oxidative stress. However, more research is needed to increase our understanding of the mechanisms that induce cardiovascular disease in OSA and so leads to new and more effective treatment modalities to prevent the cardiovascular risks associated with this increasingly common disease.

## Figures and Tables

**Table 1:** Cohort studies regarding OSA and incidence of cardiovascular disease.

Cardiovascular disease	Cohort	Sample size	Duration (years)	Findings	Reference
Hypertension	WSC	893	4 or 8	Adjusted OR of AHI ≥ 15, compared with AHI = 0 : 2.89	[4]
Coronary artery disease	SHHS	4422	8.7	Significant association only on adjusted subgroup analysis of men ≤ 70. Adjusted HR for AHI ≥ 30 compared with AHI < 5 : 1.68	[5]
Stroke	WSC	1475	4 or 8	Age and sex adjusted OR for AHI ≥ 20, compared with AHI < 5 : 4.48, nonsignificant only when adjusted to BMI.	[6]
Atrial fibrillation	Sleep-clinic patients	3542	4.7	Unadjusted HR of AHI ≥ 5, compared with AHI < 5 : 2.18	[7]

AHI: apnea-hypopnea index, BMI: body mass index, HR: hazard ratio, OR: odds ratio, SHHS: Sleep Heart Health Study, WSC: Wisconsin Sleep Cohort.

**Table 2:** Different biomarkers of oxidative stress and ROS production in rodent models of intermittent hypoxia.

Reference	Species	Hypoxia regimen	Measured marker	Result
[8]	ICR mice	8 min cycles of FIO_2_ 8.5% and 21% for 30 days	MDA	Increased
[9]	ApoE^−/−^mice	30-s cycles of FIO_2_ 6.5%–21% 8 h/day for 4 and 12 weeks	OxLDL	Increased
[10]	C57BL/6J mice	2 min 6% O_2_ and 2 min 21% O_2_ for 8 h/day for 1, 2, and 4 weeks	TBARS	Increased
[11]	CF-1 mice	30-s cycles of FIO_2 _8% 8 h/day for 21 and 35 days	DNA damage	Increased
[10]	C57BL/6J mice	2 min 6% O_2_ and 2 min 21% O_2_ for 8 h/day for 1, 2, and 4 weeks	Protein carbonyls	Increased
[12]	C57BL/6J mice	30-s cycles of FIO_2_ 4.5%–21% 8 h/day for 10 days	NADPH-dependent superoxide production	Increased
[13]	Sprague-Dawley rats	90-s cycles of FIO_2_ of 5%, 20 cycle/h, 7 h/day for 14 days	Qualitative measurement of superoxide anion	Increased
[14]	Sprague-Dawley rats	21% to 10% FIO_2_ for 5 s every 90 s for 4 weeks	SOD levels	Decreased

ApoE: apolipoprotein E, FIO2: fraction of oxygen inspired, MDA: malondialdehyde, NADPH: nicotinamide adenine dinucleotide phosphate, OxLDL: oxidized low-density lipoprotein, SOD: superoxide dismutase, TBARS: thiobarbituric acid.
